# SARS-CoV 9b Protein Diffuses into Nucleus, Undergoes Active Crm1 Mediated Nucleocytoplasmic Export and Triggers Apoptosis When Retained in the Nucleus

**DOI:** 10.1371/journal.pone.0019436

**Published:** 2011-05-27

**Authors:** Kulbhushan Sharma, Sara Åkerström, Anuj Kumar Sharma, Vincent T. K. Chow, Shumein Teow, Bernard Abrenica, Stephanie A. Booth, Timothy F. Booth, Ali Mirazimi, Sunil K. Lal

**Affiliations:** 1 Virology Group, International Centre for Genetic Engineering and Biotechnology, New Delhi, India; 2 Swedish Institute for Communicable Disease Control, Solna, Sweden; 3 The School of Biotechnology, Jawaharlal Nehru University, New Delhi, India; 4 The YLL School of Medicine, National University of Singapore, Kent Ridge, Singapore, Singapore; 5 The National Microbiology Laboratory, Public Health Agency of Canada, Winnipeg, Manitoba, Canada; 6 The Department of Medical Microbiology, University of Manitoba, Basic Medical Sciences Building, Winnipeg, Canada; University of Texas Medical Branch, United States of America

## Abstract

**Background:**

9b is an accessory protein of the SARS-CoV. It is a small protein of 98 amino acids and its structure has been solved recently. 9b is known to localize in the extra-nuclear region and has been postulated to possess a nuclear export signal (NES), however the role of NES in 9b functioning is not well understood.

**Principal Findings/Methodology:**

In this report, we demonstrate that 9b in the absence of any nuclear localization signal (NLS) enters the nucleus by passive transport. Using various cell cycle inhibitors, we have shown that the nuclear entry of 9b is independent of the cell cycle. Further, we found that 9b interacts with the cellular protein Crm1 and gets exported out of the nucleus using an active NES. We have also revealed that this NES activity influences the half-life of 9b and affects host cell death. We found that an export signal deficient SARS-CoV 9b protein induces apoptosis in transiently transfected cells and showed elevated caspase-3 activity.

**Conclusion/Significance:**

Here, we showed that nuclear shuttling of 9b and its interaction with Crm1 are essential for the proper degradation of 9b and blocking the nuclear export of this protein induces apoptosis. This phenomenon may be critical in providing a novel role to the 9b accessory protein of SARS-CoV.

## Introduction

Severe acute respiratory syndrome (SARS) was a new respiratory illness that emerged in China in 2003 and spread globally [1], [2]. The causative agent was identified as a new coronavirus and was named SARS coronavirus (SARS-CoV) [3]–[5]. The SARS-CoV genome consists of approximately 29,700 nucleotides encoding 28 putative proteins [6], [7]. Just like other coronaviruses, the SARS-CoV genome also contains several small open reading frames (ORFs) in addition to those encoding for structural proteins [6]–[9]. These small ORFs are presumed to encode 8 group specific, accessory proteins viz. ORF3a, 3b, 6, 7a, 7b, 8a, 8b and 9b [8].

One of these accessory proteins, the 9b protein is encoded by ORF-9b of the SARS-CoV genome. Just like the internal (I) gene of other group II coronaviruses, the ORF-9b of SARS-CoV overlaps with its nucleocapsid ORF [8], [10]–[12]. However, there is no homology between the SARS-CoV 9b and I protein of other coronaviruses. The 9b protein has been shown to get expressed in SARS-CoV-infected cells and antibodies against it have been found in the sera of SARS infected patients, demonstrating that the protein is produced during infection [13]–[16], but its actual function is not yet determined. Studies on 9b-structure by Meier *et al.,* (2006) revealed a 2-fold symmetric dimer having a lipid binding cavity and proposed its role in virus assembly [17]. Cellular localization of 9b has been previously reported to be predominantly cytoplasmic and membranous. Also, a nuclear export signal (NES) present in its 46-LRLGSQLSL-54 amino acid region has been suggested to be responsible for its nucleocytoplasmic export [18].

Keeping this in mind, we studied the cellular localization pattern of 9b and found that in addition to the cytoplasm, some of the 9b protein was also present in the nucleus. This entry of 9b into the nucleus was independent of cell cycle progression. Further, we showed that 9b which lacks the nuclear localization signal (NLS) continued to enter the nucleus passively and was able to exit the nucleus due to its functional NES. Also, nuclear export was found to be Crm-1 dependent and blocking NES based export resulted in an increased half-life of 9b, which accumulated in the nucleus. Finally, our studies revealed that when 9b remained within the nucleus, it triggered caspase 3 mediated apoptosis in transiently transfected mammalian cells. The requirement for caspase 3 in apoptosis induction was further confirmed using the cell permeant caspase inhibitors, Z-VAD-FMK (general caspase inhibitor) and Z-DEVD-FMK (caspase 3 inhibitor). To the best of our knowledge, this is the first report showing the nuclear localization of 9b, its passive diffusion into and active Crm-1 dependent transport out of the nucleus. Also, this is the first report associating 9b with nucleocytoplasmic export linked apoptosis.

## Materials and Methods

### Plasmids and reagents-

The SARS-CoV (GenBank accession number NC_004718) 9b gene was PCR amplified and cloned into pCDNA3.1/V5-His TOPO vector (Invitrogen) using gene specific primers; F1 (5′ GTAATGGACCCCAATCAAACCAAC 3′) and R1 (5′ TTTTGC-CGTCACCACCACGAA 3′). In order to clone 9b into pEYFPN1, it was PCR amplified using F2 (5′ CGGGAATTCCTGATGGACCCCAATCAAACC 3′) and R2 primers (5′ GTATGG- ATCCCGTTTTGCCGTCACCACCAC 3′). A mutant of 9b was made using commercially available, PCR based site directed mutagenesis services (Banglore genei, Banglore) where all leucine present in the NES region of 9b were replaced by alanine (mu-NES-9bEGFPN1: 46-**L**R**L**GSQ**L**S**L**-54 to 46-**A**R**A**GSQ**A**S**A**-54) and the mutant was further cloned into EGFPN1 vector (Clontech). Creatine phosphate, creatine phosphokinase, leptomycin B (LMB), digitonin and cell-cycle inhibitors were purchased from Sigma. Wheat germ agglutinin (WGA) was purchased from Invitrogen. Caspase inhibitors were purchased from BD Pharmingen. Cleaved caspase-3 antibody was purchased from Cell Signaling. The SARS-CoV 9b specific antibody, Alexa Fluor®488 goat-anti-rabbit IgG and Crm 1 antibodies were purchased from Abgent, Invitrogen and Abcam, respectively.

### Cell culture and transfection

Vero cells were maintained in DMEM supplemented with penicillin, streptomycin and 10% FBS. Approximately 0.6 million cells were plated in a 60 mm dish. The total amount of transfected DNA was 3 µg per 60 mm dish. The plasmid was mixed with LipofectAMINE 2000 (Invitrogen) in serum-free DMEM media (Invitrogen) and the cells were transfected as per manufacturer's protocol. For metabolic labeling, 36 hrs post-transfection, cells were starved for 1 h in cysteine/methionine-deficient medium (Invitrogen), and were then labeled with 100 µCi of [^35^S] cysteine/[^35^S] methionine promix for a specific time period. After labeling, cells were washed once in PBS and lysed in radioimmunoprecipitation assay (RIPA) buffer (150 mM NaCl, 1% NP40, 0.5% deoxycholate, 0.1% SDS and 50 mM Tris/HCl, pH 8.0 with protease inhibitor cocktail). All transfection experiments were performed in triplicates for at least three times. Standard deviation was calculated wherever needed.

### Viral Culture

Vero E6 (VE6) cells (ATCC, Global Bioresource Center) were maintained with Dulbecco's Modified Eagle Medium (DMEM) supplemented with 10% fetal bovine serum, streptomycin, penicillin and L-glutamine at 37°C and 5% CO_2_. Chamber-culture slides (BD Falcon) were seeded with Vero E6 cells to form confluent monolayers. Chambers were inoculated with SARS-CoV (Tor-3 strain) at an MOI of 5 TCID50 per cell. Infected cells were fixed in 2% paraformaldehyde in PBS, pH = 7, for 15 minutes at room temperature, at 12, 18, 24, 36 and 48 hours post infection.

### Immunofluorescence staining of SARS-CoV infected cells

Prior to use, culture slides were rinsed twice in PBS and subjected to a 10 min permeabilization step with PBS containing 0.25% Triton X-100. After washing 3×5 min in PBS and blocking with 1% (v/v) BSA in PBS containing 0.01% Tween-20 (PBST) for 1 hour at RT, slides were incubated overnight at 4°C with 1∶100 dilution of primary antibody in 1% BSA PBST (SARS virus PUP6 polyclonal, N-terminal) (Abgent). Slides were rinsed 3×5 min in PBS, and then incubated with secondary antibody, Alexa Fluor®488 goat-anti-rabbit IgG (Invitrogen) for 1 h at RT in 1% BSA PBST. After additional rinsing 3×5 min in PBS, slides were air dried and mounted in 4′,6-diamidino-2-phenylindole (DAPI) antifade reagent (Jackson ImmunoResearch), mounted with coverslips, and stored in darkness at 4°C until examination. Slides were imaged using a Zeiss LSM700 Laser Scanning Microscope with Zen2009 version 5.5 software. Three-dimensional volume projections were rendered in “Volume Viewer” using “ImageJ” version 1.43 g. Controls included mock-infected cells.

### Confocal microscopy of 9b transfected cells

The 9b transfected cells were fixed 36 hrs post-transfection in 2% paraformaldehyde (in PBS, pH = 7) for 15 min at room temperature. Paraformaldehyde was removed and cells were washed once with 1xPBS and then rehydrated in 1xPBS for 20 min. Next, the cells were mounted using antifade reagent having DAPI (Invitrogen). For localization studies, cells were imaged using LSM 510 confocal microscope (Carl-Zeiss, AG, Germany) fitted with Axiovert 200 M inverted microscope (Carl-Zeiss). Images were captured with either 63x oil objective or 40x objective using the 488 nm for GFP and 514 nm for YFP. Filter used at the detector channel for the respective proteins were BP 505–530 nm for GFP, and LP 530 nm for YFP. For (DAPI) stained nuclei, 405 laser line was used for excitation and signals were captured using LP 420 detector filter. The captured images were processed using both ‘Image J’ as well as ‘LSM image browser’ software. Approximately 200 cells were counted from each sample and protein localization was scored. Data represents analysis from 3 independent sets of experiments.

### Drug treatment

Vero cells were synchronized for 18 hrs by serum starvation. Further, these cells were transfected in duplicates with the plasmid of interest and were kept in complete media for 12 hrs. The cells were treated for 16 hrs with either nocodazole or aphidicolin (1 µg/ml and 1.5 µg/ml, respectively) and were placed in complete media for the next 24 hrs. Subsequently, one set of drug treated cells were processed for confocal microscopy and another set were used for flow cytometry experiments. For leptomycin B (LMB) treatment, cells were synchronized and were treated with 25 nM drug for 16 hrs and further incubated in complete media for another 24 hrs. For cyclohexamide treatment, transfected cells were treated with 15 µg/ml of the drug 34 hrs post-transfection. Two hours post-treatment, cells were processed for microscopy. Z-FAfmk, Z-VAD-fmk and Z-DEVD-fmk were dissolved in DMSO to give a final concentration of 50 mM and stored at -80°C until use. Immediately prior to addition to cells, the stock solutions were diluted into the culture medium.

### Propidium iodide staining and cell-cycle analysis

For propidium iodide (PI) staining, cell pellets from Vero cells were fixed in 70% ethanol at 4°C for 45 min. After being washed twice with ice-cold PBS, the cell pellet was resuspended in 500 µl PI-solution in PBS (40 µg/ml PI from 50x stock solution (2.5 mg/ml), 0.1 mg/ml RNase A and 0.05% Triton X-100) and incubated at 37°C for 30 min. For each sample, about 10,000 events were acquired using a Cyan-ADP flow cytometer (Dako, Glostrup, Denmark) at 488 nm excitation and the results were analyzed using ‘Summit (version 4.3)’ and ‘FlowJo (version 6.4)’ software. All values were expressed as mean ± SD and a paired t-test was performed.

### Fluorescent protein for *in-vitro* transport assay

Vero cells were transfected with the appropriate plasmid and were processed 36 hrs post-transfection. The cells were washed at least two times with cold phosphate buffer saline (PBS), pH 7.4, by resuspension and centrifugation, 5000 rpm, 4°C. The cells were then washed with 10 mM HEPES, pH 7.3, 110 mM potassium acetate, 2 mM magnesium acetate, 2 mM DTT and then pelleted. The cell pellet was lysed in 1.5 vol of lysis buffer (5 mM HEPES, pH 7.3, 10 mM potassium acetate, 2 mM magnesium acetate, 2 mM DTT, 1 mM PMSF, and 1 mM Na_3_VO_4_ with a protease inhibitor cocktail) (Amersham Biosciences, NJ, USA) and was processed for *in-vitro* transport assay as described by Adam *et al.,* 1990 [19].

### Cell permeablization and *in-vitro* transport assay

*In-vitro* transport assay was performed as explained by Adam *et al.,* 1990 with minor modifications [19]. Briefly, Vero cells were grown on coverslips and were permeabilized by immersion in ice cold transport buffer containing 40 µg/ml digitonin for 5 min. After 5 min, cells were washed and kept in cold transport buffer. The coverslips were then blotted to remove excess buffer and inverted over a drop of complete transport mixture on a sheet of parafilm in a humidified box. The complete transport mixture contained 50–75% fluorescent protein (freshly made as explained above) diluted with transport buffer to give the following final conditions: approx. 25–35 mg/ml fluorescent-tagged fusion protein + transport buffer (20 mM HEPES, pH 7.3, 110 mM potassium acetate, 5 mM sodium acetate, 2 mM DTT, 1.0 mM EGTA, 1 mM ATP, 5 mM creatine phosphate (Sigma), 20 U/ml creatine phosphokinase (Sigma), and protease inhibitor cocktail (Amersham Biosciences, NJ, USA)). The fluorescent protein was prepared as explained above and the entire box was then floated in a water bath at 30°C. As a positive control, we have used the NP protein of H5N1 which is known to have two NLS [20]. The 3a protein of SARS-CoV has been used as a negative control as it has been shown to localize in the extranuclear region [21]. At the end of the assay, each coverslip was rinsed and mounted in a small amount of transport buffer (without DAPI) and was observed using the confocal microscope.

### WGA treatment

Coverslips containing transfected Vero cells were incubated with transport buffer containing 50 µg/ml wheat germ agglutinin (WGA) for 15 min at room temperature. The coverslips were then blotted to remove excess buffer, inverted on a drop of complete transport mix and processed for *in-vitro* transport assay. At the end of the assay, each coverslip was rinsed and mounted in a small amount of transport buffer (without DAPI). Samples were observed by confocal microscope (Carl-Zeiss) and processed using ‘Image J’ software.

### Nuclear extract preparation and Immunoprecipitation

Vero cells were resuspended in 400 µl buffer A (10 mM HEPES pH 7.9, 10 mM KCl, 1 mM EDTA, 1 mM EGTA, 1 mM DTT, 1 mM PMSF and protease inhibitor mix). Further, the mixture was placed on ice for 15 min. After 15 min, 25 µl of 10% NP-40 was added to the cells and vortexed for 10 seconds. Cells were centrifuged at 1000 rpm for 10 min at 4°C. The supernatant was labeled as cytoplasmic fraction. The pellet was resuspended in 200 µl of buffer C (10 mM HEPES pH 7.9, 400 mM NaCl, 1 mM EDTA, 1 mM EGTA, 1 mM DTT, 1 mM PMSF and protease inhibitor mix). The samples were kept on ice for 20 min. Subsequently, the samples were centrifuged, 14000 rpm, 10 min, at 4°C and the supernatant was labeled as nuclear fraction. Immunoprecipitation was performed as described previously [22]. For SARS-CoV infected cells, nuclear and cytoplasmic fractions were prepared using CelLyticTM NuCLEARTM Extraction Kit (Sigma, MI, USA) according to manufacturer's protocol. Vero E6 cells infected by SARS-CoV (Frankfurt I strain) at MOI  = 1, were treated with or without 50 nM LMB for 24 or 48 hrs in 6 well plates. Western Blot was then performed on nuclear and cytoplasmic fractions.

### Pulse chase assay

Asynchronously growing Vero cells were transfected with appropriate constructs and incubated for 36 hrs. Subsequently, transfected or untransfected cells were pulse-labeled for 30 minutes and chased for 0, 30 and 60 minute time-points. Whole cell lysate was then prepared by cell lysis as described above in fluorescent protein preparation method. Immunoprecipitation was performed using a polyclonal anti-9b antibody and band intensities were quantified by Image J software.

### TUNEL (terminal deoxynucleotidyl transferase-mediated dUTP nick-end labelling) assay

Cell death was detected using cell-death-detection TUNEL assay kit (Roche Biochemicals), according to the manufacturer's instructions. Apoptotic cells were quantified by counting TUNEL-positive cells in each set of experiments. At least 200 cells from three separate images were inspected, and percentages were calculated.

### Apoptotic analysis

Cell extracts from transfected cells were assayed for cleaved caspase-3 by western blotting. Briefly, cell lysates were analyzed on SDS-PAGE, transferred onto nitrocellulose membrane and a western was performed using cleaved caspase-3, caspase-7 and caspase-9 specific antibodies separately (Cell signaling, USA). Band intensities were quantified using Image J software.

### Cell viability assay (MTT assay)

Vero E6 cells were infected with SARS CoV (MOI = 1) for 1 hr, then washed and treated with or without 50 nM LMB for 24 or 48 hrs. Cell viability was then determined using (3-(4,5-Dimethylthiazol-2-yl)-2,5-diphenyltetrazolium bromide (MTT) colorometric detection assay from Cell BiolabśCytoSelect TM Cell Viability and Cytotoxic Assay Kit (Cell Biolabs, Inc) according to manufacturer's protocol.

### Statistical analysis

Experiments were performed in triplicates and were repeated at least three times. Statistical analysis was done using the GRAPHPAD software. Unpaired t-test was performed and two-tailed p-value was calculated. Statistical significance of variations was considered.

## Results

### SARS-9b protein localizes in the nucleus in addition to cytoplasm

**Figure 1:**
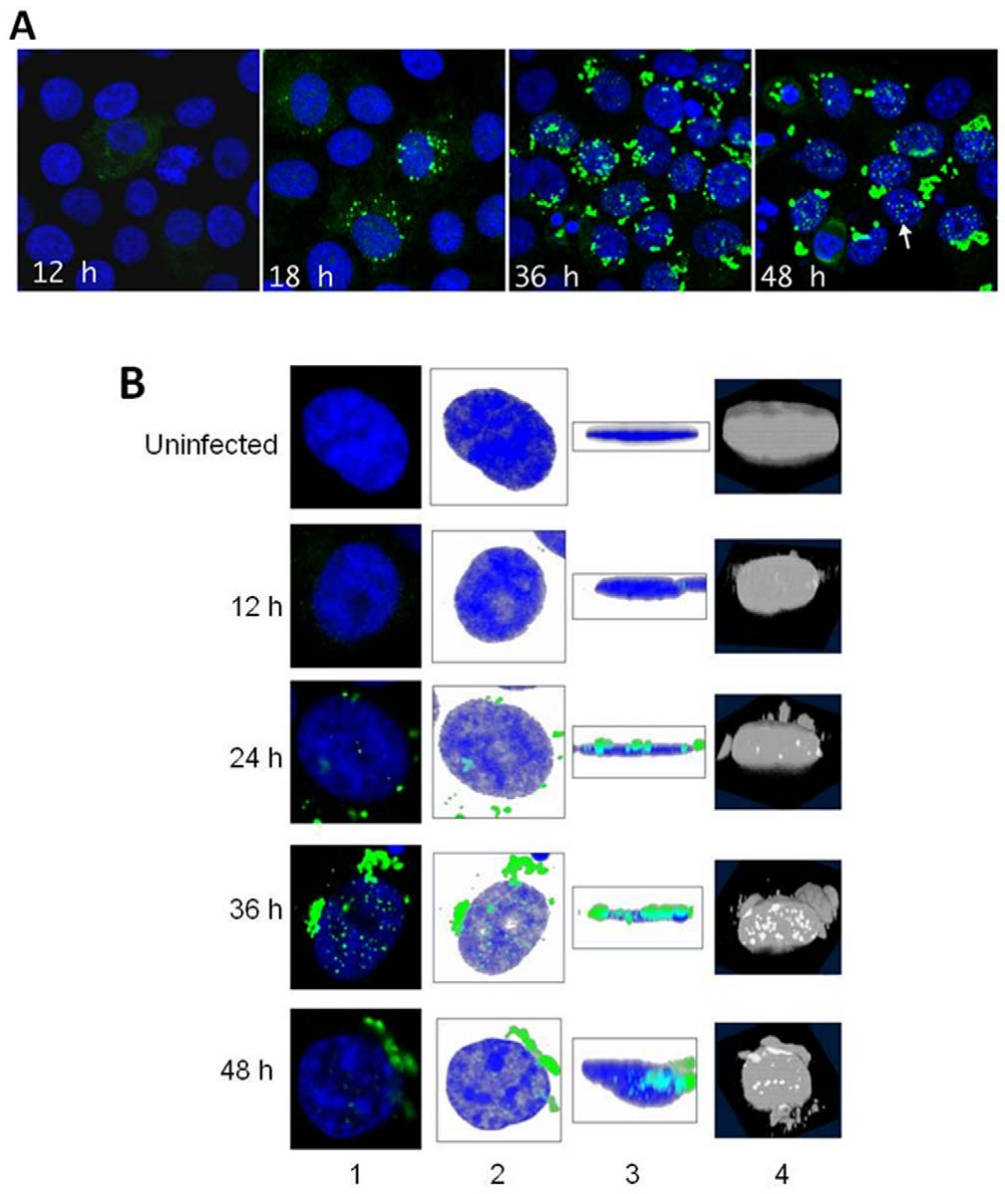
Expression of the SARS-CoV 9b protein in the cytoplasm and translocation into the nucleus in SARS-CoV infected Vero cells. A. Localization pattern of the 9b protein at various time points in SARS-CoV infected cells. Chamber-culture slides (BD Falcon) were seeded with Vero E6 cells to form confluent monolayers. Chambers were inoculated with SARS-CoV (Tor-3 strain) at an MOI of 5 TCID50 per cell. Infected cells were fixed in 2% paraformaldehyde in PBS, pH = 7, for 15 minutes at room temperature, at 12, 18, 24, 36 and 48 hours post infection. Slides were imaged using a Zeiss LSM700 Laser Scanning Microscope with Zen2009 version 5.5 software. Three-dimensional volume projections were rendered in “Volume Viewer” using “ImageJ” version 1.43 g. Figure shows merged 2-channel confocal images of SARS-CoV infected Vero E6 cells stained with anti-9b antibodies (green: Alexa Fluor 488) and DAPI (blue), imaged at 12, 18, 36 and 48 h post-infection under a 63 x oil immersion lens. Nuclear inclusions of 9b are numerous at 36 and 48 h post-infection (an example is arrowed). B. Two and three-dimensional confocal imaging of the time course of infection with SARS-CoV labelled with antibody for 9b (green) and DAPI counter-stain for nuclei (blue). The first column shows zoomed close-ups of typical examples of an uninfected cell, and infected cells at 12, 24, 36, and 48 h post-infection respectively. Column 2 shows projections of three-dimensional reconstructions, using maximum intensity in the xy orientation (looking from the top view), made with the images taken of the cells shown in column 1. Column 3 is a projection of the three-dimensional reconstruction in the zx orientation (side view). Column 4 shows sections through the three-dimensional reconstructions of the nucleus, viewed at a 45 degree angle to the culture surface. The bright spots in the nucleus are the Alexa-labelled 9b antibody positive regions within the nucleus, clearly indicating that these are within the nuclear membrane.

Cells infected with SARS-CoV showed faint cytoplasmic staining with antibodies directed at the 9b protein at 12 h post-infection (Fig. 1a). By 18 h post-infection, many discrete cytoplasmic foci were observed, and by 24 h post-infection, small foci of nuclear staining appeared, while control uninfected cells showed no staining (Fig. 1b). Typical infected cells showed numerous nuclear foci of 9b protein by 36 h post-infection, as well as extensive, larger areas of cytoplasmic inclusions (Fig. 1a and 1b). By 48 h post-infection, many nuclei were seen to be changing their appearance and undergoing apoptosis, and majority of the cells at this time point contained fewer foci of the 9b protein (Fig. 1b, 48 h time point, second panel). Stacks of confocal images were used to generate three-dimensional projections, with merged data from the alexa fluor and DAPI channels (Fig. 1b). When viewed from the side and from the top, as well as sections through the volume projections, it was observed that the antibody staining (in green) was within the nucleus as well as the cytoplasm. These foci of fluorescence first appeared in the nucleus at 24 h post infection to reach a maximum at about 36 hr. p.i., and are somewhat reduced in numbers at 48 h p.i. (Fig. 1b). After the observation that some proportion of the 9b protein enters into the nucleus, we planned to check its localization in 9b transfected cells. Vero cells were transfected with the plasmid constructs pEYFPN1-9b (having 9b sequence fused to a yellow fluorescent protein coding sequence at the C-terminus region). Analysis of 9b-YFP localization showed that in addition to the extra-nuclear region, some amount of 9b was also present within the nucleus similar to the SARS-CoV infected cells (Fig. S1, panel (i), (ii) and (iii)). In a parallel experiment, only pEYFPN1 vector was transfected in order to rule out any effect due to the vector itself (Fig. S1, panel (iv), (v) and (vi)). Only cells showing clear nuclear localization of 9b were scored positive. Similar results were also obtained with HEK-293T and COS-7 cells (data not shown).

**Figure 2:**
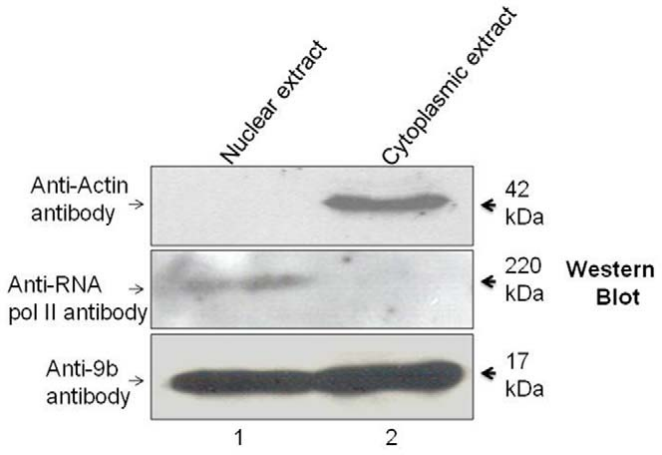
The SARS-CoV 9b protein localizes in both cytoplasm as well as nucleus when expressed in transfected cells. Vero cells were transfected with pCDNA3.1/V5-His TOPO-9b construct. After 36 hrs, cells were processed for nuclear and cytoplasmic extract preparation. These extracts were used for western blotting using appropriate antibodies. Lane 1 represents nuclear extract and lane 2 represents cytoplasmic extract. Lowermost panel shows that 9b was present both in cytoplasm as well as nucleus. Upper and central panel shows the purity of the cytoplasmic and nuclear extracts respectively.

In order to biochemically confirm the nuclear localization of the 9b protein, we transfected Vero cells with pCDNA3.1-V5 His-9b construct. After 36 hrs, cells were processed for nuclear and cytoplasmic extract preparation. As shown in Figure 2, 9b was present both in cytoplasm as well as nucleus. The purity of the extracts was checked using anti-actin and anti-polymerase II antibodies for the cytoplasmic and nuclear extracts, respectively (Fig. 2, upper and central panel).

### SARS-9b localization pattern is independent of cell-cycle progression

**Figure 3:**
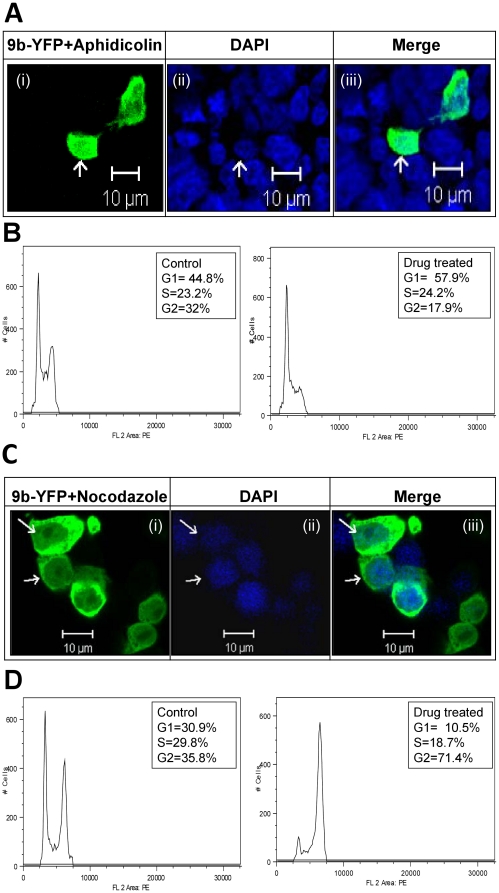
Cell-cycle inhibitors do not affect the localization pattern of the SARS-CoV 9b protein. Cells were synchronized in duplicates by starvation and were transfected with pEYFPN1-9b. Drug treatment was given. One set was processed for microscopy and the other set was used for flow cytometric analysis. **A.** The effect of aphidicolin treatment on 9b protein localization pattern. Synchronized pEYFPN1-9b transfected cells were treated with 1.5 µg/ml aphidicolin (Sigma). Yellow color of 9b was converted into green. Panel (i), (ii) and (iii) shows 9b, DAPI and a merge image respectively. Arrow shows nuclear entry of 9b. **B.** The efficacy of aphidicolin was confirmed by flow cytometeric analysis. Left histogram shows control cells and right histogram shows drug treated cells. Aphidicolin inhibited the S-phase entry of cells. **C.** The effect of nocodazole treatment on 9b protein localization pattern. Synchronised cells were treated with 1 µg/ml nocodazole (Sigma). Yellow color of 9b was converted into green. Panel (i), (ii) and (iii) shows 9b, DAPI and a merge image respectively. Arrow shows nuclear entry of 9b. **D.** The efficacy of nocodazole was confirmed by flow cytometeric analysis. Nocodazole treated cells were stained with PI and analyzed by flow cytometer. Left histogram shows control cells and right histogram shows drug treated cells. Nocodazole inhibited the G2-M phase entry.

The cellular localization of many proteins is cell-cycle specific [23]–[25]. In order to examine the effects of cell-cycle on 9b localization, we blocked cells either in the G1 or G2 stage, treating them with different cell-cycle inhibitors. For G1 phase, the cells were treated with aphidicolin (G1-S phase inhibitor) at a concentration of 1.5 µg/ml and processed for microscopy and flow cytometry analysis. The microscopy results clearly showed that the cellular localization pattern of 9b remained unchanged in the presence of aphidicolin (Fig. 3a). Flow cytometer analysis was done to confirm the efficacy of the drug. The percentage of cells arrested at G1-S phase of the cell-cycle after aphidicolin treatment were found to be 57.9% [±0.794%] as compared to untreated cells (44.8%±0.361%) confirming a significant arrest at this phase (p = 0.0015) (Fig. 3b). Blocking cell-cycle at the G2 phase (using nocodazole) did not affect the cellular localization pattern of the 9b protein, indicating that there was no effect of cell-cycle progression on the cellular localization and nuclear transport of the 9b protein (Fig. 3c). Flow cytometry analysis showed that the percentage of cells arrested at the G2-M phase of the cell-cycle after nocodazole treatment was 71.4% [±2.689%] as compared to untreated cells (35.8%±1.453%) showing significant cell-cycle arrest at this phase (p = 0.0027) (Fig. 3d).

### SARS-9b protein lacks an active NLS and enters the nucleus by passive mode of transport

**Figure 4:**
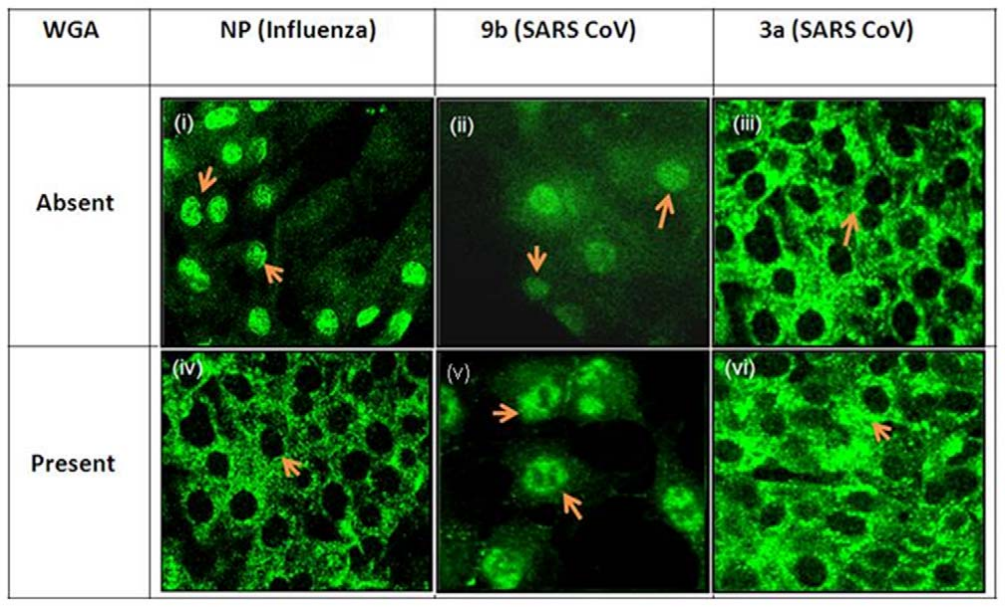
The SARS-CoV 9b protein enters the nucleus by using passive mode of transport. **Upper panels**: An *in-vitro* transport assay to confirm the nuclear localization of SARS-CoV 9b protein. An *in-vitro* transport assay performed in absence of WGA shows nuclear localization of SARS-CoV 9b protein. Panel (i) shows the localization of GFP tagged nucleocapsid (NP) protein of influenza (having NLS) that was used as a positive control in this assay. Arrow shows the nuclear localization of NP protein. Panel (iii) shows the GFP tagged SARS-CoV 3a protein that was used as a negative control for the *in-vitro* transport assay. Arrows clearly show that the 3a protein is not able to enter the nucleus. Panel (ii) shows that even in *in-vitro* transport assay, SARS-CoV 9b protein localizes in both cytoplasm as well as nucleus. Cytoplasmic 9b protein was somewhat diffused which may be explained as the effect of digitonin permeablization. Arrows shows 9b protein localization in digitonin permeablized cells. **Lower panels:** An *in-vitro* transport assay for 9b protein performed in the presence of WGA. The *in-vitro* transport assay performed in presence of 50 µg/ml WGA shows a passive mode of import for SARS-CoV 9b protein. Panel (iv) shows that the nucleocapsid protein of influenza was unable to enter the nucleus in presence of WGA showing that WGA was able to block the NLS dependent active transport into the nucleus. As shown in panel (vi), the SARS-3a protein was unable to get into the nucleus in WGA treated, digitonin permeablized cells. As shown in panel (v), the SARS-CoV 9b protein was able to enter the nucleus even in the presence of WGA showing that its entry is independent of active transport pathway. Arrows in various panels show the localization of protein.

Cellular localization of some well known proteins like p27Kip1, transcription factor lymphoid enhancer factor 1 (LEF-1), T-cell factor 1 (TCF-1), cystinosin and some viral proteins have been confirmed by *in-vitro* transport assays using digitonin-permeabilized cells [26]–[29]. To investigate the mode of nuclear transport of 9b, an *in-vitro* transport assay was performed. Initially, the 9b-YFP fusion protein was expressed in Vero cells separately and then their lysate was added to digitonin permeabilized cells. The cells were supplied with all necessary components needed for *in-vitro* transport as detailed in materials and methods. Results showed that both H5N1-NP (a positive control in the assay) and 9b-YFP were able to enter the nucleus in digitonin permeabilized cells (Fig. 4, panel (i) and (ii), respectively). On the contrary, the 3a-GFP protein (negative control) was not able to get into the nucleus (Fig. 4, panel (iii)). A protein sorting signal analysis of the 9b sequence (using PSORT program, http://psort.org/) predicted absence of NLS in it and suggested its nuclear import to be passive [30]. Further, in order to rule out the possibility that the 9b protein uses an active transport mode to enter the nucleus, we used wheat germ agglutinin (WGA), a lectin known to inhibit NLS dependent active nuclear transport [31]. When WGA was added to the cells, H5N1-NP (known to undergo NLS dependent nuclear import) was found to get restricted to cytoplasm indicating that WGA was able to inhibit the active transport of H5N1-NP protein through the nuclear membrane (Fig. 4, panel (iv)). However, SARS-CoV 9b showed similar nuclear localization even in the presence of WGA (Fig. 4, panel (v)). Thus, our data clearly shows that although the SARS-CoV 9b protein lacks a NLS, it enters the nucleus by passive transport. As expected, the negative control, SARS-3a remained cytoplasmic (Fig. 4, panel (vi)).

### SARS-9b protein gets exported from the nucleus using the Crm-1 dependent NES

**Figure 5:**
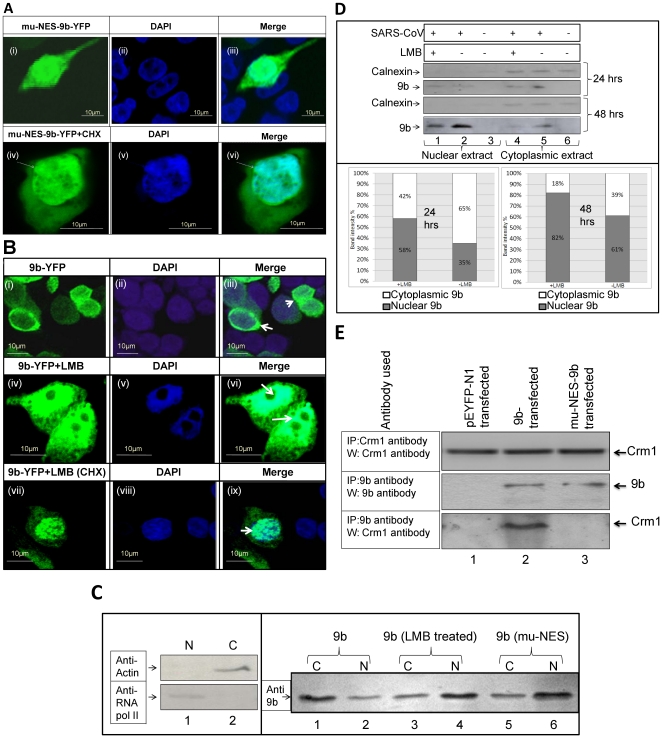
The nuclear export of 9b is active and Crm-1 dependent. **A.** The localization pattern of SARS-CoV 9b protein having mutated NES. The localization of SARS-CoV 9b mutant (having alanine at place of leucine) show that in presence of mutated NES, the protein gets concentrated in nucleus. Panel (i), (ii) and (iii) shows mu-NES-9b, DAPI and a merge image respectively. In order to reduce the background resulting from newly synthesized 9b protein, transfected cells were treated with cyclohexamide for two hours and were processed for microscopy. Panel (iv), (v) and (vi) shows the protein localization in cyclohexamide treated cells. Arrow shows nuclear accumulation of 9b. **B.** The localization pattern of SARS-CoV 9b protein in the presence of LMB. Synchronized pEYFPN1-9b transfected Vero cells were treated with 25nM LMB. Results show that in presence of the drug used, most of the 9b protein gets blocked in the nucleus. Yellow color of 9b was converted into green. Panel (i), (ii) and (iii) shows 9b, DAPI and a merge image respectively. Panel (iv), (v) and (vi) shows the protein localization in LMB treated cells. Panel (vii), (viii) and (ix) shows the effect of cyclohexamide (CHX) on LMB treated cells. Arrow shows the localization of 9b. **C.** Biochemical analysis of the localization pattern of either mutated SARS-CoV 9b protein or wild type SARS-CoV 9b protein in the presence of LMB. Synchronized cells were transfected with the appropriate constructs. Nuclear and cytoplasmic extracts were prepared 36 hrs post 9b transfection and were ran on a 15% SDS-PAGE gel followed by a western blot using anti-9b polyclonal antibody. Left panel shows purity of extracts. Results in right panel show that both mu-NES-9b as well as LMB treated 9b have increased nuclear localization of 9b. C and N represent cytoplasmic and nuclear extracts respectively. **D.** Biochemical analysis of the localization pattern of 9b protein in SARS-CoV infected cells. Synchronized cells were infected with the virus. Nuclear and cytoplasmic extracts were prepared 24 and 48 hrs post infection and were analyzed on 15% SDS-PAGE gel followed by western blotting using appropriate antibody. The upper panel shows that 9b is present in both nuclear as well as cytoplasmic extracts. Calnexin was used to check the purity of extracts. Lower panel shows the densitometric comparison of nuclear and cytoplasmic 9b using Image J program. Results show that LMB treatment (50 nM) leads to an increased nuclear localization of 9b as compared to untreated cells. **E.** The SARS-CoV 9b protein interacts with Crm-1. Synchronized cells were transfected with the appropriate constructs and the lysate was immunoprecipitated using the mentioned antibodies. Next, western blotting was performed using either anti-9b or anti-Crm1 antibodies as per the case. Lane 2 in the lowermost panel clearly shows that 9b is able to pull out the endogenous Crm-1 protein. However, mutated NES clearly shows inhibition of binding of 9b with Crm-1 further confirming the presence of NES in 46–54 amino acid region. Lane 1 represents pEYFP-N1 vector which has been used as a negative control. Uppermost and central panel shows the expression level of Crm-1 and 9b respectively in the transfected cells. IP represents immunoprecipitation; W represents western blotting.

The nuclear export signal (NES) has been reported to be present in the C-terminal region of the 9b protein [18]. To confirm the involvement of the NES in 9b export, we mutated the essential amino-acids of the NES region (46-**L**R**L**GSQ**L**S**L**-54 to 46-**A**R**A**GSQ**A**S**A**-54) and cloned it into the pEGFPN1 vector (now onwards called as mu-NES-9b). Cells were transfected with this mutant and were analyzed 36 hrs after transfection. Microscopic analysis clearly revealed that most of the fluorescence accumulated in the nucleus (Fig. 5a, panel i-iii). Further, in order to check the role of NES in nuclear export of 9b, we treated the 9b-EYFPN1 transfected cells with Leptomycin-B (LMB); a drug known to inhibit active NES based export [32], [33]. We found that most of the 9b protein accumulated within the nucleus upon LMB treatment, confirming the role of NES in its nuclear export (Fig. 5b, panel iv–vi). In order to reduce the cytoplasmic staining resulting from the newly synthesized 9b protein, transfected cells were treated with cyclohexamide and processed for microscopy. The reduction in cytoplasmic fluorescence in lower panels of Figure 5A and 5B confirms the effect of cyclohexamide treatment (Fig. 5a, panel iv–vi and Fig. 5b, panel vii–ix). The data obtained was confirmed by performing biochemical analysis of the fractionated lysates of transfected cells. Nuclear and cytoplasmic extracts were prepared 36 hrs post-transfection and were analyzed on 15% SDS-PAGE gel followed by a western blot using anti-9b polyclonal antibody. Purity of the extracts was checked as described before. Results confirmed our previous observations (Fig. 5c). Taken together, all these results proved that 9b follows a NES dependent, active nuclear export. In order to confirm the localization pattern of 9b in the presence or absence of LMB, SARS-CoV infected cells were processed for western blotting at two different time points, 24 hrs and 48 hrs. Calnexin was used as purity control for nuclear and cytoplasmic extract preparation. The 9b protein was seen in both nucleus as well as cytoplasm at both 24 hrs and 48 hrs (lane 2 and 5, Fig. 5d, upper panel). This shows that 9b localizes both in the nucleus as well as in the cytoplasm in both transfection and infection conditions. Further, the effect of LMB was checked and the proportion of 9b in nuclear and cytoplasmic extracts was compared in reference to total protein using densitometric analysis (Fig. 5d, lower panel). As shown in Figure 5d, total 9b in LBM treated cells seem slightly lower compared to untreated cells. This is most probably due to more cells in apoptotic phase. However, densitometry graphs clearly show that the percentage of nuclear 9b gets increased on LMB treatment which further supports our transfection data. The effect seems more pronounced at the 48 hrs time point. The only difference we observed between transfection and infection conditions was that even without LMB, a lot of 9b was seen in the nucleus 48 hrs post infection. This may be attributed to the impact of other SARS-CoV proteins (expressed during infection) on 9b localization.

Active nuclear export takes place by interaction of the nuclear export signal (NES) with a protein, known as Crm-1 and is critical for RNA and protein export from the nucleus [32], [33]. Synchronized cells were transfected with the appropriate constructs and the lysate was immunoprecipitated using the mentioned antibodies. The complex was subjected to SDS-PAGE followed by western blotting using either anti-9b or anti-Crm1 antibodies. As a control, the levels of Crm-1 were measured by performing immunoprecipitation using anti-Crm-1 antibody followed by western blotting with the same antibody. Results show an interaction of 9b with the Crm-1 protein, clearly supporting our previous observations (Fig. 5e, lane 2). Further, mu-NES-9b was unable to bind Crm-1, confirming the presence of NES activity in the 46 to 54 amino-acid region (Fig. 5e, lane 3).

### NES dependent nuclear export facilitates intracellular degradation of the 9b protein

**Figure 6:**
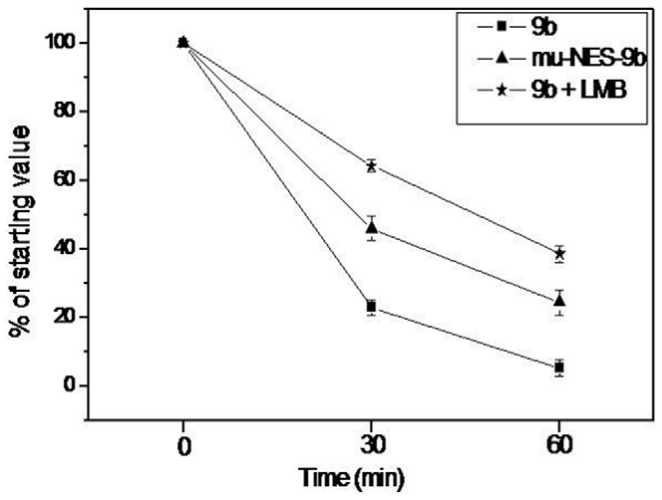
Nuclear export of SARS-CoV 9b protein promotes its degradation. Pulse chase analysis of 9b protein shows that the half life of 9b-EYFPN1 was approximately 20 minutes in cell culture. Export-deficient 9b mutant showed a significant increase in half-life of 9b. Similar results were observed upon inhibition of 9b export by LMB. Band intensities from three independent experiments were quantified using a phosphoimager and graphed with standard errors for 9b-EYFPN1, LMB treatment as well as mu-9b NES. The p value for differences between 9b, mu-NES-9b and 9b+LMB was calculated using “Graphpad” software and ranged from p = 0.0004 to p = 0.0013.

Many cellular proteins are known to follow the ubiquitin/proteasome pathway for their degradation [34]. For shuttle proteins, nuclear export is known to promote degradation [35]. Our data has already indicated that 9b behaves like a shuttle protein, going into the nucleus passively and coming out using the NES. Keeping these facts in mind, we designed experiments to investigate whether nuclear export influences the intracellular stability of 9b protein. Vero cells transiently expressing 9b-EYFPN1 or mu-NES-9b were metabolically pulse-labeled followed by a chase period for 0, 30 and 60 min. Compared to 9b, we observed a significantly prolonged half-life for mu-NES-9b, indicating that nuclear export is continuously supplying substrate for the degradation machinery. Similar results were observed for 9b-EYFPN1 transfected cells treated with a nuclear export inhibitor, LMB (Fig. 6). The experiment was performed in triplicates and the significance of the data was calculated using “Graphpad” software. The differences between 9b, mu-NES-9b and 9b+LMB for time point of 30 min and 60 min were significant as the p value ranged from p = 0.0004 to p = 0.0013.

### Blocking active nuclear export of 9b protein induces apoptosis in transfected cells

**Figure 7:**
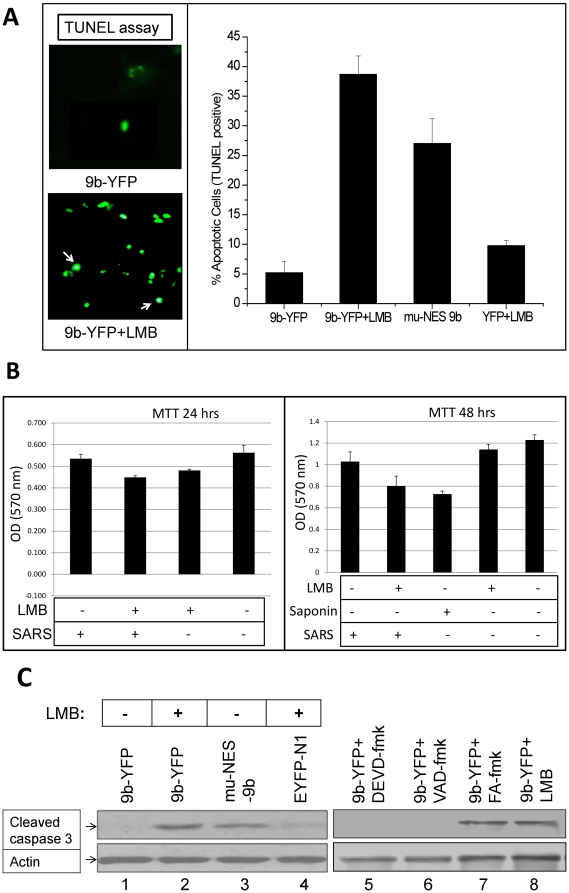
Inhibition of nuclear export of SARS-CoV 9b protein induces apoptosis. Vero cells were transfected with appropriate plasmid. In order to chemically block active export, cells were treated with LMB for 16 hrs. **A.** The SARS-CoV 9b protein induces apoptosis, if blocked in the nucleus. A TUNEL assay was conducted in order to quantify the apoptotic cells. TUNEL-positive cells were counted from a total sample size of 200 cells in each set of experiments. Standard deviation was calculated from three independent experiments. The graph shows percentage of apoptotic cells. Arrows in left panel shows TUNEL positive cells. **B.** MTT assay was performed to check the effect of LMB (50 nM) on cell viability in SARS-CoV infected cells. Saponin was used as control. Maximum viability was lost when SARS CoV infected cells were treated with LMB. **C.** Apoptosis induced by the nuclear blocked SARS-CoV 9b protein is caspase 3 dependent. Cell lysate was used to measure cleaved caspases by Western blotting. Among all, only cleaved caspase 3 was picked up demonstrating the role of caspase 3 mediated apoptosis in cells having export deficient 9b protein. Lane 2 and 3 clearly shows that the cleaved caspase 3 is present only in LMB treated 9b protein or NES mutant of 9b respectively. Lane 1 and lane 4 shows the controls used in the assay. A faint band for cleaved caspase 3 was also seen in lane 4 which is attributed to the effect of drug treatment. To confirm that the apoptosis is caspase dependant, caspase inhibitors like Z-VAD-fmk and Z-DEVD-fmk (200 µM each) were added to cells 16 hr post transfection, and then every 4 h thereafter for a total of four doses. The cells were lyzed 36 hrs post transfection and the lysates were checked for the levels of cleaved caspase-3. Both Z-DEVD-fmk and Z-VAD-fmk were able to inhibit apoptosis (lane 5 and lane 6). The negative control inhibitor (Z-FA-fmk) had no inhibitory effect on apoptosis (lane 7). Results confirm that apoptosis induced by 9b (if blocked in the nucleus) is caspase dependent.

During our pulse-chase analysis explained above, it was discovered that a number of 9b-EYFPN1 transfected cells died on treatment with LMB. For quantification of the apoptotic cells, TUNEL assay was performed (Fig. 7a, left panel). The number was significantly higher for LMB treated 9b transfected cells as compared to untreated cells. Similar results were observed for mu-NES-9b (Fig. 7a, right panel). These results indicated that blocking 9b nuclear export may induce cell death by apoptosis. Similar results were obtained when we checked the cell viability in SARS-CoV infected cells by MTT assay. Maximum viability of SARS-CoV infected cells was lost on LMB treatment suggesting that 9b nuclear export may be important for cell survival (Fig. 7b). However, the role of other viral proteins in this process can not be ignored. Saponin was used as control. To check the pathway thus involved in induction of apoptosis, we determined the levels of various cleaved caspases (caspase 3, 7 and 9) in transiently transfected cells. Western blot results show that 9b-EYFP induce apoptosis (only if its nuclear export is blocked) by increasing the cellular levels of cleaved caspase-3 (Fig. 7c, lanes1–4). As expected, mu-NES-9b induced apoptosis even in the absence of LMB. To confirm that the apoptosis is caspase dependant, Z-FA-fmk, Z-VAD-fmk and Z-DEVD-fmk (200 µM each) were added to cells 16 hrs post-transfection, and then every 4 hrs thereafter, for a total of four doses. The lysates were checked for the levels of cleaved caspase-3. Results confirmed that apoptosis induced by 9b (blocked in the nucleus) was caspase dependent (Fig. 7c, lanes 5–8). All these results collectively showed that the export deficient 9b protein lead to caspase-3 dependent apoptosis of the host cells. Therefore, an NES based nuclear export of 9b appears to be very significant for host survival and may play a vital role in supporting the SARS-CoV life cycle.

## Discussion

### The SARS-CoV 9b protein passively diffuses into the nucleus and undergoes active Crm-1 mediated nucleocytoplasmic export

The present study uncovers an important step in understanding the roles of accessory proteins in the SARS-CoV life-cycle. In this study, we characterized the nucleocytoplasmic shuttling of the 9b protein and found that 9b enters the nucleus by a passive mode of transport whereas its export is active and NES-Crm1 interaction dependent. Further, in transfection experiments, we showed that this localization pattern of 9b was independent of cell-cycle stages. A time course study of 9b revealed that the pattern of its cellular localization remained unchanged irrespective of post-transfection time (data not shown). Disruption of 9b-Crm1 binding after mutating the critical residues within the NES, and nuclear accumulation of 9b on treatment with LMB, a Crm1 antagonist, reconfirmed our hypothesis.

Earlier studies on 9b show that it localizes in the cytoplasm [17], [18]. We also found the cytoplasmic localization of 9b, however our biochemical as well as confocal analysis clearly showed that 9b, in addition to cytoplasm, also localizes in the nucleus. This import is not active and is NLS independent as 9b is able to enter into the nucleus even in the presence of WGA. WGA is known to bind to the nuclear pore complex and inhibit the nuclear localization signal (NLS)-dependent intracellular transport [8], [36]. Proteins below 50 kDa have been reported to get into the nucleus passively [37], [38]. SARS-CoV 9b is a small protein of about 13 kDa, thus there is a fair possibility of passive diffusion of 9b even as a dimer. However, the possibilities of exposure of a hidden NLS after conformational changes cannot be ignored. Our preliminary data suggests that 9b interacts with many nuclear proteins (Fig. S2A–C). These interactions may also provide a possible reason for the presence of 9b in the nucleus.

Cell-cycle inhibitors are known to affect the cellular localization of some proteins [24], [25], [39]. To confirm the effect for 9b, we used various cell-cycle inhibitors. Aphidicolin, arrests G1-S phase progression of the cell-cycle by specially inhibiting DNA polymerase α, responsible for DNA replication [25], [40], [41]. Nocodazole is an anticancer drug that interferes with the structure and function of microtubules during interphase and blocks cell-cycle progression at the G2-M phase [41]. Our results show that the localization of 9b is independent of cell-cycle stages and the pattern of distribution of 9b remains unaltered in the presence of the drugs used. A low level of apoptosis observed for nocodazole treated cells is a well established effect of this drug [42].

LMB inhibits NES dependent protein export [32], [33]. The suggested mechanism involves direct binding of LMB to Crm1 (exportin 1), which blocks the binding of Crm1 to proteins containing the NES. Deletion of the NES present in 9b as well as LMB treatment of cells has been reported to retain 9b in the nucleus [18]. We observed similar results when we repeated these experiments. Further when we mutated the essential amino-acids within the NES by site-directed mutagenesis or used cells treated with LMB; we found accumulation of 9b in the nucleus. We performed a BLAST analysis of the NES region of SARS-CoV 9b protein which revealed that this region was well conserved (except the replacement of Q by N at amino-acid position 51 in a few cases) irrespective of the host of the virus.

### NES dependent nuclear export facilitates intracellular degradation of the 9b protein

Interestingly; this phenomenon of nuclear transport exhibited by 9b is similar, but not identical to some other known proteins. Proteins like MAPK and survivin are reported to localize in both cytoplasm as well as the nucleus [43], [44]. Similar to 9b protein, survivin protein does not have NLS but contains NES. Survivin also enters passively into the nucleus. However, blocking nuclear export of survivin enhances its half-life [44]. Our results show that the 9b behaves similar to the survivin protein. We found that the half-life of 9b protein in cells is about 20 minutes and an active nuclear export is mandatory for proper degradation of the 9b protein. Our pulse-chase analysis clearly shows an approximate two-fold increase in the half-life of 9b protein, when its export from nucleus is blocked. The dependence of 9b degradation on NES is also supported by increased half-life of both; mutant (lacking essential amino acids of its NES region) as well as 9b treated with LMB. The NES based nuclear export has been shown to promote degradation of shuttle proteins like nuclear factor kappa B or inhibitory kappa B by the proteasome or ubiquitin pathway [35]. Similar to 9b, the degradation of many shuttle proteins has been reported to get delayed if their export is inhibited [44].

### The SARS-CoV 9b protein triggers caspase 3 mediated apoptosis when retained in the nucleus of mammalian cells

While performing pulse-chase assays, we found that a significant number of Vero E6 cells, in which nuclear export of 9b has been inhibited (either by treating with LMB or using NES deficient 9b), were showing caspase 3 dependent apoptosis. The absence of significant cell death in case of EYFP-N1 transfected, LMB treated cells, ruled out the possibility of drug induced cell death. All these results indicate that 9b if blocked in the nucleus, activates apoptotic signaling pathway/s. However, the possibilities for this observation to be cell-type specific can not be ignored.

**Figure 8:**
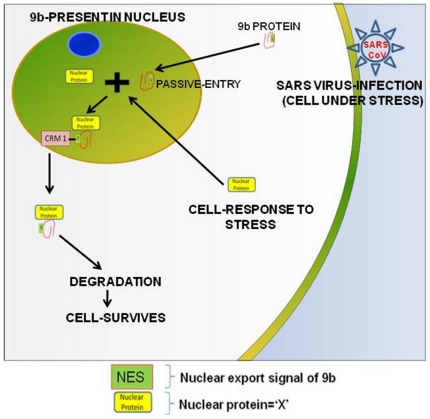
Hypothesis showing probable role of 9b localization. There are certain proteins which function as transcription factor and on activation (by stress or infection) move into the nucleus. Nuclear localization of such proteins further regulates the genes that play a role in apoptosis and cell cycle progression. We propose that NES of 9b may remain unexposed until it binds to the nuclear proteins (represented as ‘nuclear protein’ = ‘X’). Our preliminary data shows that 9b binds with the nuclear proteins (Fig. S2). This interaction may be exposing NES of 9b leading to the export of ‘X’ proteins along with 9b using Crm1 dependent pathway. This event may stop host cell response against stress caused by the viral infection.

Summing up, we propose a hypothesis describing the possible role of the SARS-CoV 9b protein in maintaining the cell survival after viral infection (Fig. 8). There are certain proteins which function as transcription factor and on activation (by stress or infection) move into the nucleus [45], [46]. Nuclear localization of such proteins further regulates the genes that play a role in apoptosis and cell-cycle progression. We propose that the NES of 9b may remain unexposed until it binds to the nuclear proteins. Our preliminary results show that 9b binds with the nuclear proteins (Fig. S2). These interactions may be exposing the NES of 9b leading to the export of ‘X’ proteins along with 9b using the Crm1 dependent pathway. This event may stop host cell response against stress caused by the viral infection.

Recent studies on accessory proteins of coronaviruses including SARS-CoV reveal that these proteins are not essential for virus replication and pathogenecity [47]–[49], but studies are there to support the fact that accessory proteins do affect virus release, stability, pathogenesis, and finally contribute towards virulence [50]. In MHV, I protein (an accessory viral structural protein) can contribute to plaque morphology [10]. Also, SARS-CoV (9b protein) has been shown to be a structural component of the virions which again indicates the importance of 9b protein for the virus [51]. However, more studies need to be performed on the functional analysis of the 9b protein.

Thus, besides other mechanisms like dimerization, lipid binding or interaction with other proteins, export of 9b seems to be a very important phenomenon for both functional activities of 9b as well as the SARS-CoV life-cycle. *In-vivo* studies as well as identification of the nuclear protein/s specifically binding to 9b will help in further understanding the role of 9b in nucleocytoplasmic shuttling. While it is not sure whether SARS-CoV will again emerge in the human population, it has spurred on the awareness against it. Hopefully, this study will add a step towards the better understanding of the behavior and function of the 9b accessory protein in the life-cycle of the SARS-CoV.

## Supporting Information

Figure S1 **Microscopy results show that the SARS-9b protein localizes in both cytoplasm as well as nucleus when expressed in transfected mammalian cells.** Vero cells were transfected with either pEYFPN1-9b or pEYFPN1 alone. After 36 hrs, cells were fixed and mounted. Arrow in panel (i) indicates that some protein also enters into the nucleus. Panel (iv) shows the localization pattern of pEYFPN1 vector as a control. Panel (ii) and (v) corresponds to the DAPI staining of panel (i) and (iv) respectively. Panel (iii) and (vi) show a merge image. Arrows in various panels show nucleus of the cell.
(TIF)

Figure S2 **SARS-CoV 9b pulls down some specific proteins from nuclear extract of Vero cells.** **A.** The pCDNA3.1/V5-His TOPO-9b was used for *in-vitro* transcription and translation. When ran on a 15% SDS-acrylamide gel, dried and processed by autoradiography, a band of approx. 17 kDa was seen on the autoradiogram (lane 2). Lane 1 shows the mock lysate. **B.** The TNT expressed 9b protein was immunoprecipitated using anti-9b specific antibody (Abgent). The antibody was able to recognize the 9b protein (lane 2). M represents mock lysate. **C.** Vero cells were processed and the nuclear proteins were extracted as explained in material and methods. The TNT expressed 9b protein was added to the nuclear extract and a pull down assay was performed using 9b specific antibody (Abgent). In parallel, one control reaction having nuclear extract incubated with the TNT product of an empty pCDNA 3.1 vector (labeled as mock lysate) was also assembled. The pulled-out proteins were run on a 15% SDS PAGE followed by Coomassie blue staining. Lane 1 shows the proteins pulled out with 9b protein. Lane 2 shows the proteins pulled out with mock lysate. Lane 3 shows a pull-down using a non-specific antibody. The protein ladder is shown in lane 4. N.E. represents nuclear extract. N.S. represents non-specific. Arrow indicates the 9b protein on the gel.
(TIF)
