# Supplementary figures and images for: SARS-CoV 9b Protein Diffuses into Nucleus, Undergoes Active Crm1 Mediated Nucleocytoplasmic Export and Triggers Apoptosis When Retained in the Nucleus
Source: PLoS One. 2011 May 27;6(5):e19436. doi: 10.1371/journal.pone.0019436 (PMC3103500; doi:10.1371/journal.pone.0019436)

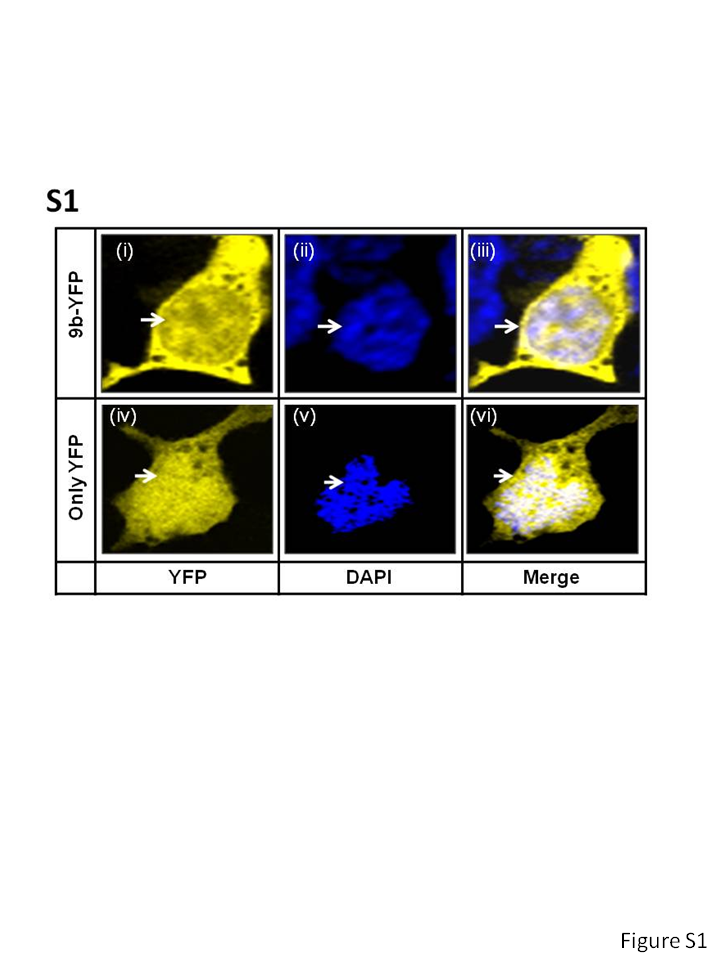

Supplement: Figure S1 — Microscopy results show that the SARS-9b protein localizes in both cytoplasm as well as nucleus when expressed in transfected mammalian cells. Vero cells were transfected with either pEYFPN1-9b or pEYFPN1 alone. After 36 hrs, cells were fixed and mounted. Arrow in panel (i) indicates that some protein also enters into the nucleus. Panel (iv) shows the localization pattern of pEYFPN1 vector as a control. Panel (ii) and (v) corresponds to the DAPI staining of panel (i) and (iv) respectively. Panel (iii) and (vi) show a merge image. Arrows in various panels show nucleus of the cell. (TIF) [file pone.0019436.s001.tif]

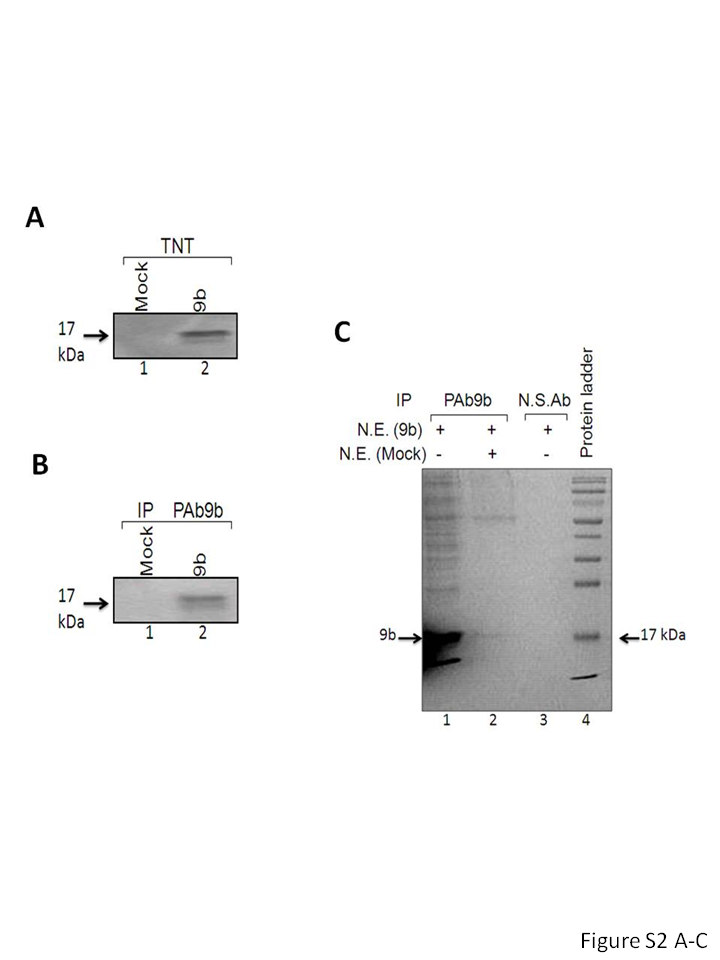

Supplement: Figure S2 — SARS-CoV 9b pulls down some specific proteins from nuclear extract of Vero cells. A. The pCDNA3.1/V5-His TOPO-9b was used for in-vitro transcription and translation. When ran on a 15% SDS-acrylamide gel, dried and processed by autoradiography, a band of approx. 17 kDa was seen on the autoradiogram (lane 2). Lane 1 shows the mock lysate. B. The TNT expressed 9b protein was immunoprecipitated using anti-9b specific antibody (Abgent). The antibody was able to recognize the 9b protein (lane 2). M represents mock lysate. C. Vero cells were processed and the nuclear proteins were extracted as explained in material and methods. The TNT expressed 9b protein was added to the nuclear extract and a pull down assay was performed using 9b specific antibody (Abgent). In parallel, one control reaction having nuclear extract incubated with the TNT product of an empty pCDNA 3.1 vector (labeled as mock lysate) was also assembled. The pulled-out proteins were run on a 15% SDS PAGE followed by Coomassie blue staining. Lane 1 shows the proteins pulled out with 9b protein. Lane 2 shows the proteins pulled out with mock lysate. Lane 3 shows a pull-down using a non-specific antibody. The protein ladder is shown in lane 4. N.E. represents nuclear extract. N.S. represents non-specific. Arrow indicates the 9b protein on the gel. (TIF) [file pone.0019436.s002.tif]
